# Tolerogenic Vaccination Reduced Effector Memory CD4 T Cells and Induced Effector Memory Treg Cells for Type I Diabetes Treatment

**DOI:** 10.1371/journal.pone.0070056

**Published:** 2013-07-19

**Authors:** Jingyao Zhang, Wenjuan Gao, Xu Yang, Jingjing Kang, Yongliang Zhang, Qirui Guo, Yanxin Hu, Guoliang Xia, Youmin Kang

**Affiliations:** 1 State Key Laboratory for Agro-Biotechnology, College of Biological Science, China Agricultural University, Beijing, China; 2 Department of Modern Sciences & Technology, Agricultural University of Hebei, Baoding, China; 3 College of Veterinary Medicine, China Agricultural University, Beijing, China; McGill University Health Center, Canada

## Abstract

**Background:**

Vaccination could induce immune tolerance and protected NOD mice from the development of type I diabetes (T1D). We previously demonstrated that insulin peptide (B9-23) combined with dexamethasone (DEX) stimulated the expansion of antigen specific regulatory T (Treg) cells which in turn effectively prevented T1D in NOD mice. Here, we aimed to investigate the therapeutic effect of tolerogenic vaccination for T1D treatment.

**Methodology/Principal Findings:**

The diabetic NOD mice (Blood glucose level ≧250 mg/dl) were treated with B9-23 and DEX twice. The tolerance was restored by blocking maturation of dendritic cells (DCs) and inducing Treg cells in treated NOD mice. Remarkably, the reduction of autoreactive effector memory CD4 T (Tm) cells and the induction of functional effector memory Treg (mTreg) cells contributed to the improvement of T1D in treated NOD mice.

**Conclusions/Significance:**

Tolerogenic vaccination restored tolerance and ameliorated T1D by suppressing effector CD4 Tm cells and inducing effector mTreg cells. Our findings implicate the potential of tolerogenic vaccination for T1D treatment.

## Introduction

T1D results from a chronic destruction of insulin-producing β cells, presumably mediated by autoreactive CD4 T cells [1]. Interventions are less effective on activated T cells, including Tm cells in pancreatic islets, as the pathogenic response becomes established [2]. Autoreactive T cells are important mediators of T1D and have been shown to be antigen-specific Tm cells targeting islet antigen in T1D patients [3]. Self-antigen specific Tm cells were observed in diabetic patients, but not in healthy individuals [4]. When naive T lymphocytes are antigen activated, the expressions of several adhesion and homing molecules increase or decrease, leading to an activated effector memory cell phenotype of CD44^High^CD62L^Low^ [5]. In T1D mice, islet-infiltrating cells were characterized as CD44^High^CD62L^Low^ which appeared to be memory cells and able to transfer insulitis and diabetes [6]. Using MHC class II tetramers, autoantigen-specific CD4 Tm cells are prevalent in the early progression to T1D [7]. In this study, CD44^High^CD62L^Low^ cells were used as markers of effector Tm cells in T1D mice.

More than 400 agents or agent combinations have been investigated in preclinical T1D, such as cyclosporine, anti-CD3 antibody for T cells or anti-CD20 antibody for B cells, and TNF-α or IL-1 blocking agents. These agents broadly inhibit the immune response. However, responses to infections could be inappropriately suppressed [8]. The self-antigen induced Treg cells have been shown potential in maintaining immunological self-tolerance as prevention or therapy for autoimmune diseases [2], [9]. The expression of transcription factor Foxp3 and cytokine IL-10 play critical roles in suppressive function of Treg cells [10], [11]. The deliberate induction of Tregs has generally been difficult to achieve *in vivo*, and there is a pressing need to develop effective methods for generating Tregs in a predictable way.

Vaccination with autoreactive antigen or peptides could suppress the immune response by inducing Treg cells for prevention or therapy of autoimmune disease[12]–[14]. Several vaccination strategies using islet antigens had been shown to modify the time of onset and severity of T1D in mice [15]. When incomplete Freund’s adjuvant (IFA) was co-administered with insulin peptides subcutaneously, T1D development was inhibited. However, when it was given intraperitoneally, the disease was not modified [16]. Co-immunization with insulin and DNA encoding proinsulin induced CD4^+^CD25^–^ islet-specific Treg cells and prevented T1D onset [17]. Our previous study demonstrated that tolerogenic vaccination, insulin peptide B9-23 combined with dexamethasone (DEX), could induce antigen specific Treg cells and effectively prevented development of T1D [18].

Currently, there is no effective treatment strategy to preserve residual β-cells and restore tolerance for T1D [8]. Therefore, there is an immediate need to restore both β cell function and immune tolerance to control disease progression and ultimately cure T1D. Based on these observations, we sought to investigate the therapeutic effect of tolerogenic vaccination on T1D treatment. Our results demonstrate that tolerogenic treatment restored tolerance and ameliorated T1D by reducing CD4 Tm cells and producing activated and memory T regulatory (mTreg) cells. These results suggest a new method for T1D treatment.

## Results

### Therapeutic Effect of Tolerogenic Treatment on T1D

**Figure 1:**
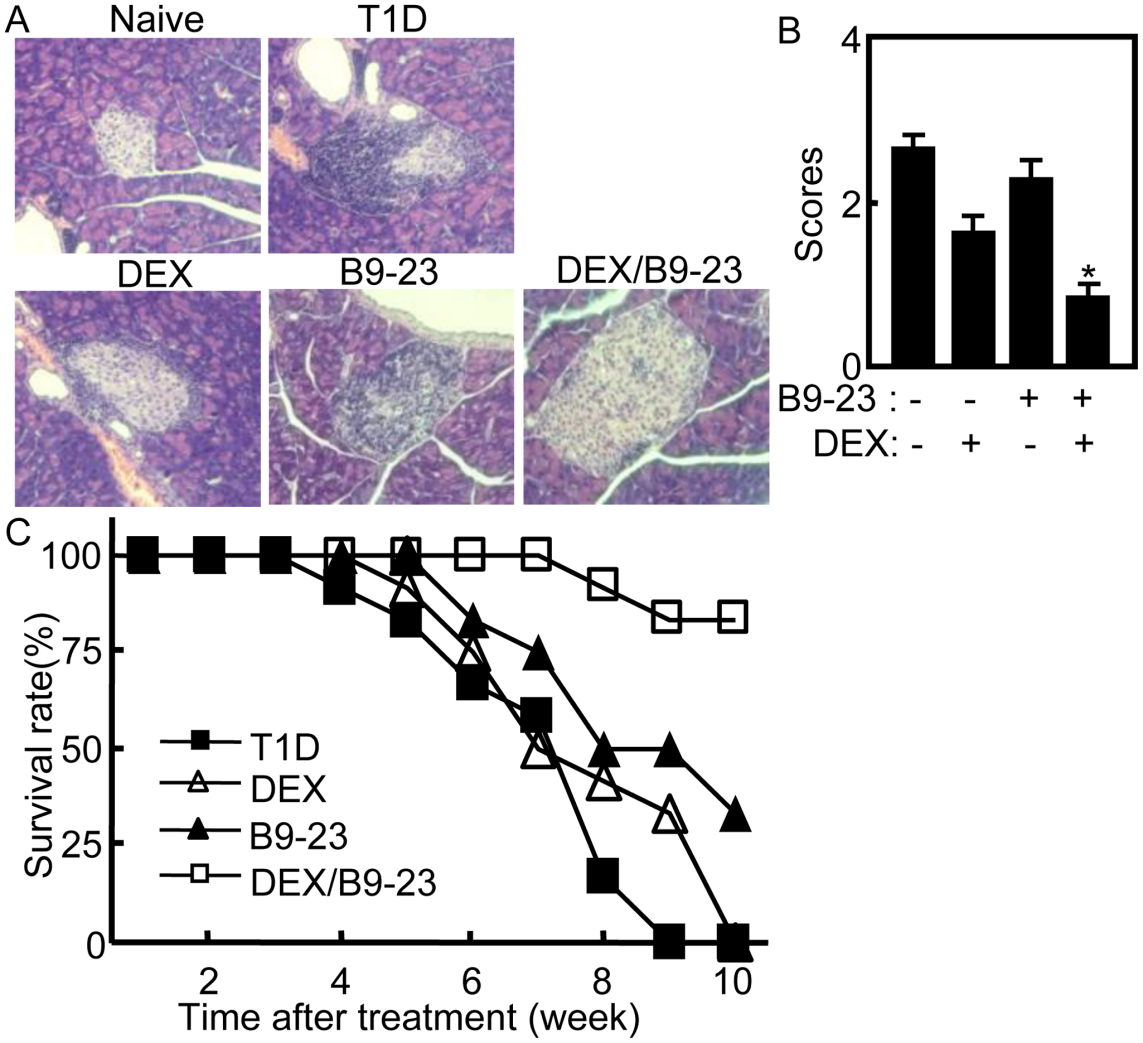
Tolerogenic treatment ameliorated T1D. A. On day 7 after the second treatment, pancreases of treated NOD mice were collected and fixed in 4% formaldehyde for 24 h before being embedded in paraffin. Serial sections were cut and stained with H&E (200×). Data shown are representative of 3 independent experiments. B. Pancreatic sections from each group were scored for islet inflammation. Shown is the average of three independent experiments with similar results. For statistical analysis, mice treated with B9-23/DEX were compared with other control groups and ANOVA were used; *p<0.05. C. The survival diabetic mice were counted weekly after treatment. Shown is the summary of three independent experiments with similar results. For statistical analysis, mice treated with B9-23/DEX were compared with other control groups and ANOVA were used; *p<0.05.

Vaccination with autoantigen or peptides could induce tolerance and effectively prevented autoimmune diseases [13], [15], [18]. We previously demonstrated that B9-23 combined with DEX could effectively prevented T1D in NOD mice [18]. To investigate the therapeutic effect of this method, the diabetic NOD mice were treated with B9-23/DEX twice. On day 7 after the second treatment, the pancreases were prepared for histological section. The lowest level of infiltration was noted in pancreas of mice treated with B9-23/DEX compared with other groups. The highest level of infiltration was observed In T1D control group, as compared to mice treated with DEX, B9-23, or B9-23/DEX (Figure 1A). Since DEX has immunosuppressive function, the infiltrated lymphocytes in pancreas of DEX treated mice were less than that in T1D control group (Figure 1A). For insulitis, the lowest score was shown in B9-23/DEX treated mice compared with that in other control groups (*p*<0.05). The score of insulitis in T1D control mice was higher than that in other groups. The score of insulitis in DEX treated mice was lower than that in T1D control and B9-23 treated mice (Figure 1B). The treated diabetic mice were monitored and counted weekly for survival rate analysis. On 6 weeks after treatment, statistical analysis of survival diabetic mice in B9-23/DEX group reached significant difference compared with that in other groups (Figure 1C). The treated mice were also monitored for glycemia analysis. There were no significant differences of glycemia level among all groups since T1D was developed (Data not shown). These results demonstrate that B9-23/DEX treatment ameliorated T1D and improved the survival time of diabetic mice.

### Restoration of Immune Tolerance in Treated Diabetic Mice

**Figure 2:**
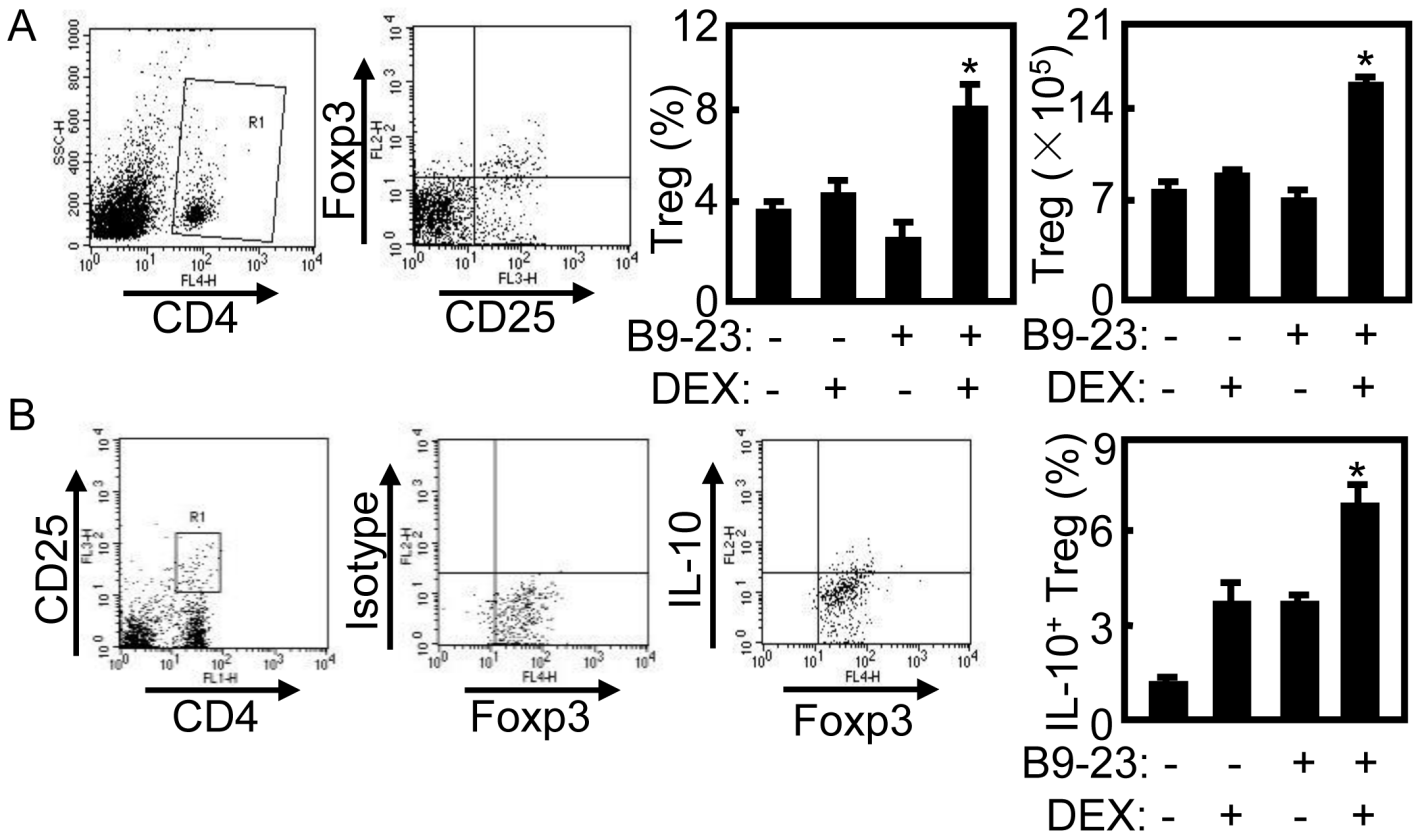
Tolerance was restored in treated diabetic mice. A. On day 7 after the second immunization, splenocytes were intracellularly stained with anti-CD4-APC, anti-CD25-PECy5 and anti-Foxp3-PE mAbs for Treg analysis. Gating on CD4^+^ T cells (R1), Treg cells (CD4^+^CD25^+^Foxp3^+^) were quantified relatively to total CD4^+^ T cells. The numbers of Treg cells were counted in all groups by flow cytometry. For statistical analysis, mice treated with B9-23/DEX were compared with other control groups and ANOVA were used; *p<0.05. B. On day 7 after the second immunization, splenocytes were re-stimulated with B9-23, and then intracellularly stained with anti-CD4-FITC, anti-CD25-PECy5, anti-Foxp3-PE and anti-IL-10-APC mAbs for IL-10 expression in Treg cells analysis. Gating on CD4^+^ CD25^+^ T cells (R1), Treg cells expressed IL-10 (CD4^+^CD25^+^Foxp3^+^ IL-10^+^) were quantified relatively to total Treg cells. Data shown are representative of 3 independent experiments. Bar, mean and SD from 2-4 independent experiments, each using at least three mice per group (n = 3). For statistical analysis, mice treated with B9-23/DEX were compared with other control groups and ANOVA were used; *p<0.05.

Many studies confirm the function of Treg cells in suppressing pathologic immune responses of autoimmune diseases [2]. Treg cells had been induced under certain therapeutic interventions in autoimmune disease [19], [20]. Our previous study demonstrated that tolerogenic vaccination could expand Treg cells in nondiabetic NOD mice and prevented T1D of NOD mice [18]. To test whether tolerogenic vaccination can induce tolerance for T1D treatment, the splenocytes of treated diabetic mice were prepared and stained with anti-CD4 and anti-CD25 mAbs, then were intracellularly stained with anti-Foxp3 mAb for Treg cells. For IL-10 expression in Treg cells, the samples were stained with anti-CD4 and anti-CD25 mAbs, then were intracellularly stained with anti-Foxp3 mAb and anti-IL-10 mAbs. Gating on CD4^+^ T cells (Figure 2A), the percentage of Treg cells (CD4^+^CD25^+^Foxp3^+^) was counted relatively to total CD4 T cells. As shown in Figure 2A, the number and percentage of Treg cells were increased significantly in B9-23/DEX treated mice compared with that in other groups (*p*<0.05). Furthermore, the percentage of IL-10^+^ Treg cells (CD4^+^CD25^+^ Foxp3^+^IL-10^+^) to total Treg cells (CD4^+^ CD25^+^ T cells, R1 in Figure 2B) was increased significantly in B9-23/DEX treated mice compared with that in other groups (*p*<0.05, Figure 2B). These results suggest that Treg cells could be induced and played suppressive function by expressing IL-10 in B9-23/DEX treated diabetic mice.

**Figure 3:**
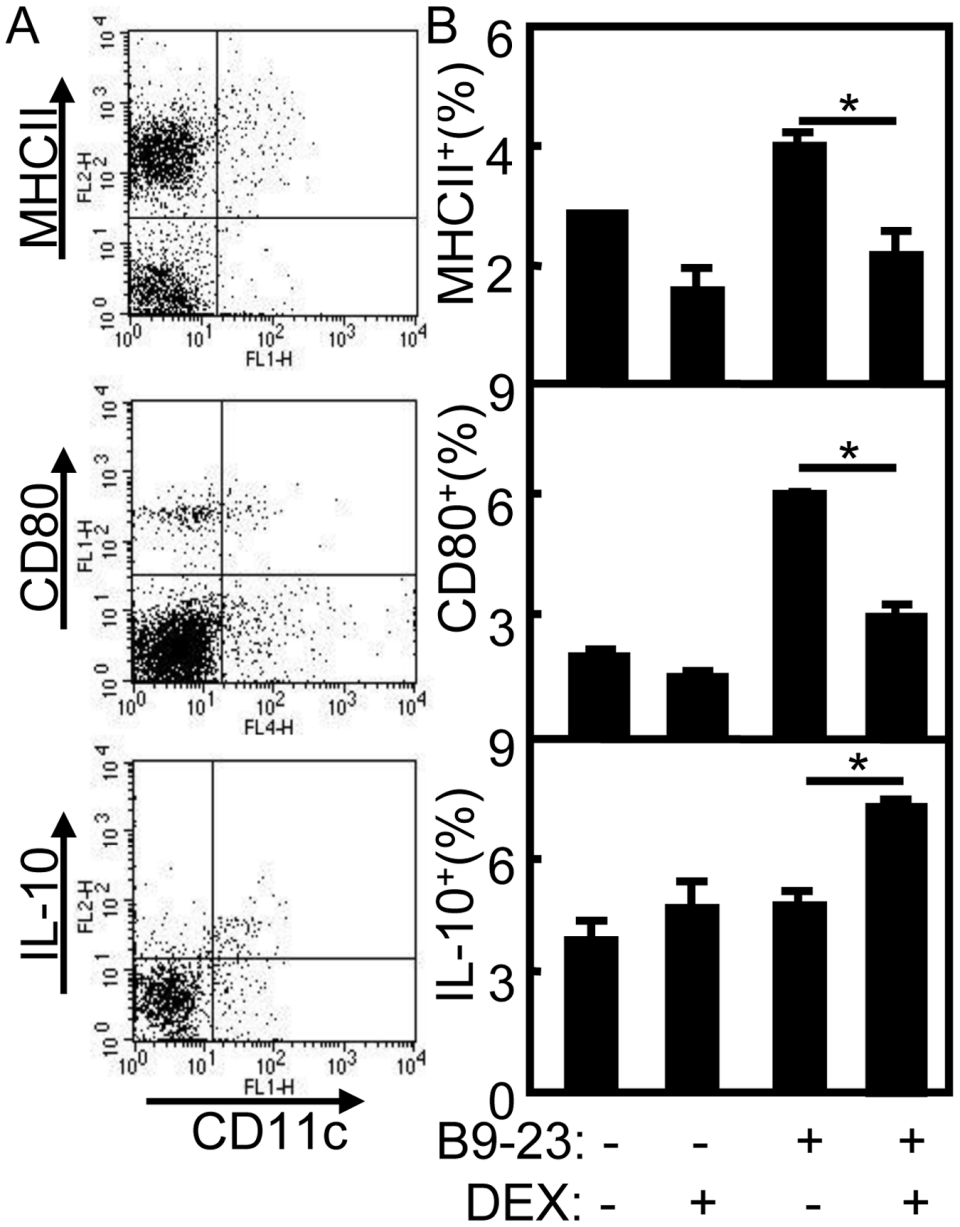
Blockage of DCs maturation in treated diabetic mice. A. On day 3 after the second immunization, the splenocytes were prepared and stained with anti-CD11c-FITC, anti-MHCII-PE or CD80-PE for DCs maturation. For IL-10^+^ DCs, the samples were stained with anti-CD11c-FITC, fixed, permeabilized, and intracellularly immunostained with anti-IL-10-PE mAbs. CD11c^+^MHCII^+^ (Upper), CD11c^+^CD80^+^(Middle), CD11c^+^IL-10^+^ cells (Lower) were counted relatively to total CD11c^+^ DC cells by flow cytometry. B. The summary of CD11c^+^MHCII^+^ (Upper), CD11c^+^CD80^+^ (Middle), CD11c^+^IL-10^+^ cells (Lower). Bar, mean and SD from 3 independent experiments, each using at least three mice per group (n = 3); For statistical analysis, mice treated with B9-23/DEX were compared with that in mice treated with B9-23 group and student-t test were used between the indicated pair; *p<0.05.

Treg cell expansion is known to be linked to the function of immature DC [19], [21], and DEX was previously reported to prevent DC maturation in vitro [22]. To analyze the maturation of DCs in treated diabetic mice, the splenocytes of treated mice were prepared and stained with anti-CD11c-FITC, anti-MHCII-PE or CD80-PE. For IL-10 expression in DCs, the samples were intracellularly stained with anti-CD11c-FITC and anti-IL-10-PE mAbs and analyzed by flow cytometry (Figure 3A). As shown in Figure 3B, the expression of MHCII or CD80 on DCs were decreased significantly in B9-23/DEX treated diabetic mice compared with that in B9-23 treated mice (*p*<0.05). The expression of CD40 or CD86 on DCs were also decreased significantly in B9-23/DEX treated diabetic mice compared with that in B9-23 treated mice (Data not shown). The percentage of IL-10^+^ DCs was increased significantly in B9-23/DEX treated diabetic mice compared with that in other control groups (*p*<0.05). These results indicate that DCs maturation was blocked in B9-23/DEX treated diabetic mice.

### Reduction of Pancreatic CD4 T Cells in Treated Diabetic Mice

**Figure 4:**
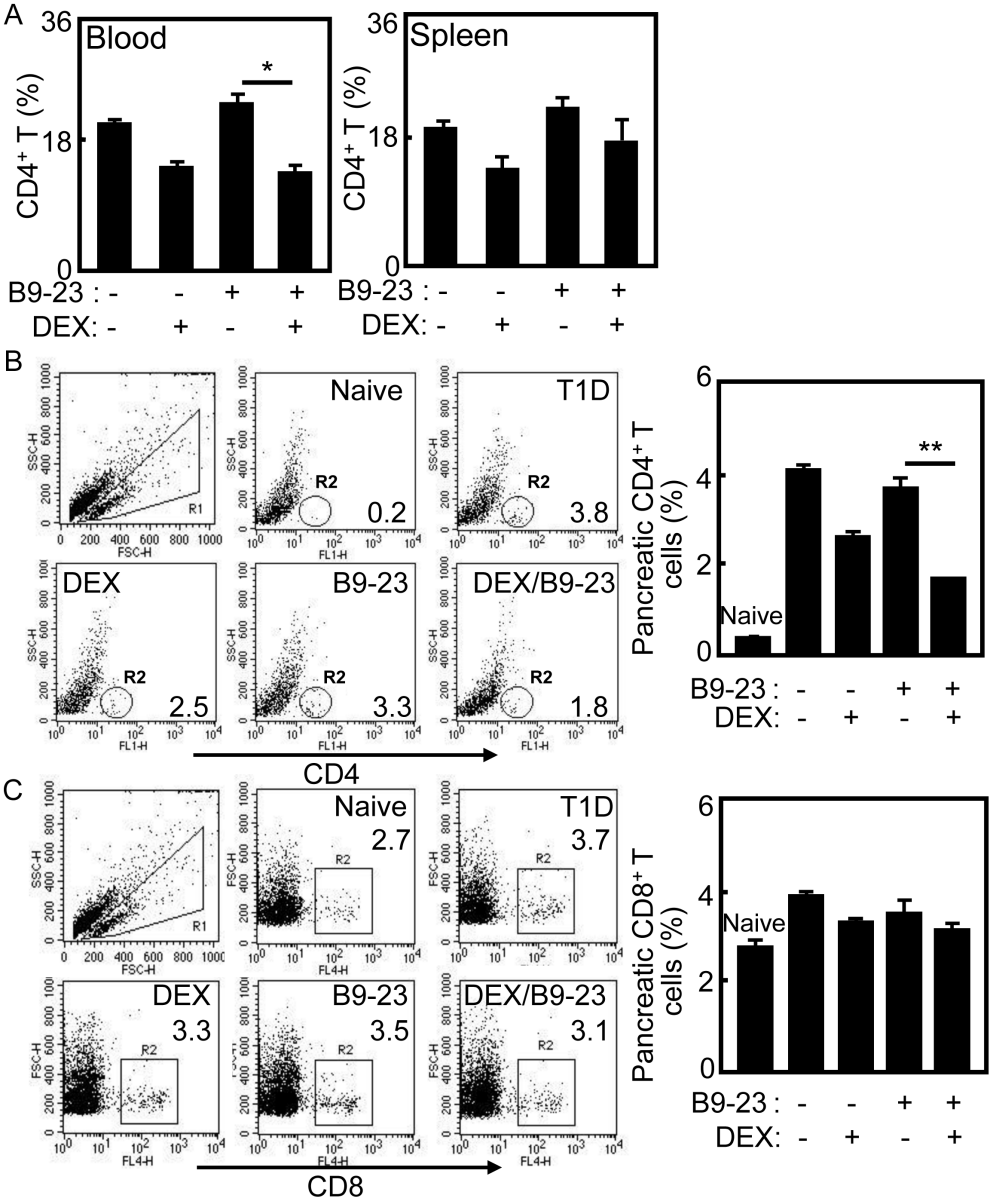
Autoreactive CD4 T cells suppressed in treated mice. A. On day 7 after the second immunization, the PBMC and splenocytes were prepared and immunostained with anti-CD4 mAb. The samples were stained with anti-CD4-FITC mAb and analyzed by flow cytometry. Data shown are representative of 3 independent experiments. Bar, mean and SD from 3 independent experiments, each using at least three mice per group (n = 3). For statistical analysis, mice treated with B9-23/DEX were compared with that in mice treated with B9-23 group and student-t test were used between the indicated pair; *p<0.05. B. On day 7 after the second immunization, the pancreatic samples were immunostained with anti-CD4-FITC mAb and analyzed by flow cytometry. The infiltrated lymphocytes (R1) were gated for CD4 T cells analysis. The CD4 T cells (CD4^+^, R2) were counted relatively to the infiltrated lymphocytes. Data shown are representative of 3 independent experiments. Data shown are representative of 3 independent experiments. Bar, mean and SD from 3 independent experiments, each using at least three mice per group (n = 3). For statistical analysis, mice treated with B9-23/DEX were compared with that in mice treated with B9-23 group and student-t test were used between the indicated pair; **p<0.01. C. On day 7 after the second immunization, the pancreatic samples were immunostained with anti-CD8-APTC mAb and analyzed by flow cytometry. The infiltrated lymphocytes (R1) were gated for CD8 T cells analysis. The CD8 T cells (CD8^+^, R2) were counted relatively to the infiltrated lymphocytes.

Cell mediated immunity plays a central role in autoimmune responses and also contributes to the destruction of insulin producing β cells in NOD mice and T1D patients [23]–[25]. To test the subpopulations of T cells in B9-23/DEX treated mice, the blood, spleen and pancreas were prepared for flow cytometry analysis. As shown in Figure 4A, the percentage of CD4 T cells in blood was lowered significantly in B9-23/DEX treated diabetic mice compared with that in B9-23 treated mice (*p*<0.05), but there were no differences of CD4 T cells in blood between B9-23/DEX and DEX treated mice. In spleens, there were no differences of CD4 T cells among all groups. Remarkably, the percentage of infiltrated CD4^+^ T cells in pancreas was decreased significantly in B9-23/DEX treated diabetic mice compared with other groups (*p*<0.05) (Figure 4B). Since DEX has immunosuppressive function, the percentage of CD4^+^ T cells in blood and spleen of DEX treated mice was decreased compared with T1D control or B9-23 groups. The numbers of infiltrated pancreatic CD4 T cells in T1D control and B9-23 treated mice were higher than that in DEX and B9-23/DEX treated groups. In B9-23/DEX treated group, there was the least number of infiltrated CD4 T cells in the pancreas than that in other groups except naïve mice (data not shown).

Pathogenic CD8 T cells can recognize β cell autoantigens and play an important role in destruction of islet in T1D patients or mice [26], [27]. To test the role of CD8 T cell population in tolerogenic treatment, the blood, splenic and pancreatic samples were stained for flow cytometry analysis. There were no differences of CD8 T cells in blood and spleen among all groups (data not shown), and the same as the pancreatic CD8 T cells (Figure 4C).

### Decrease of Autoreactive CD4 Effector Tm Cells in Treated Diabetic Mice

**Figure 5:**
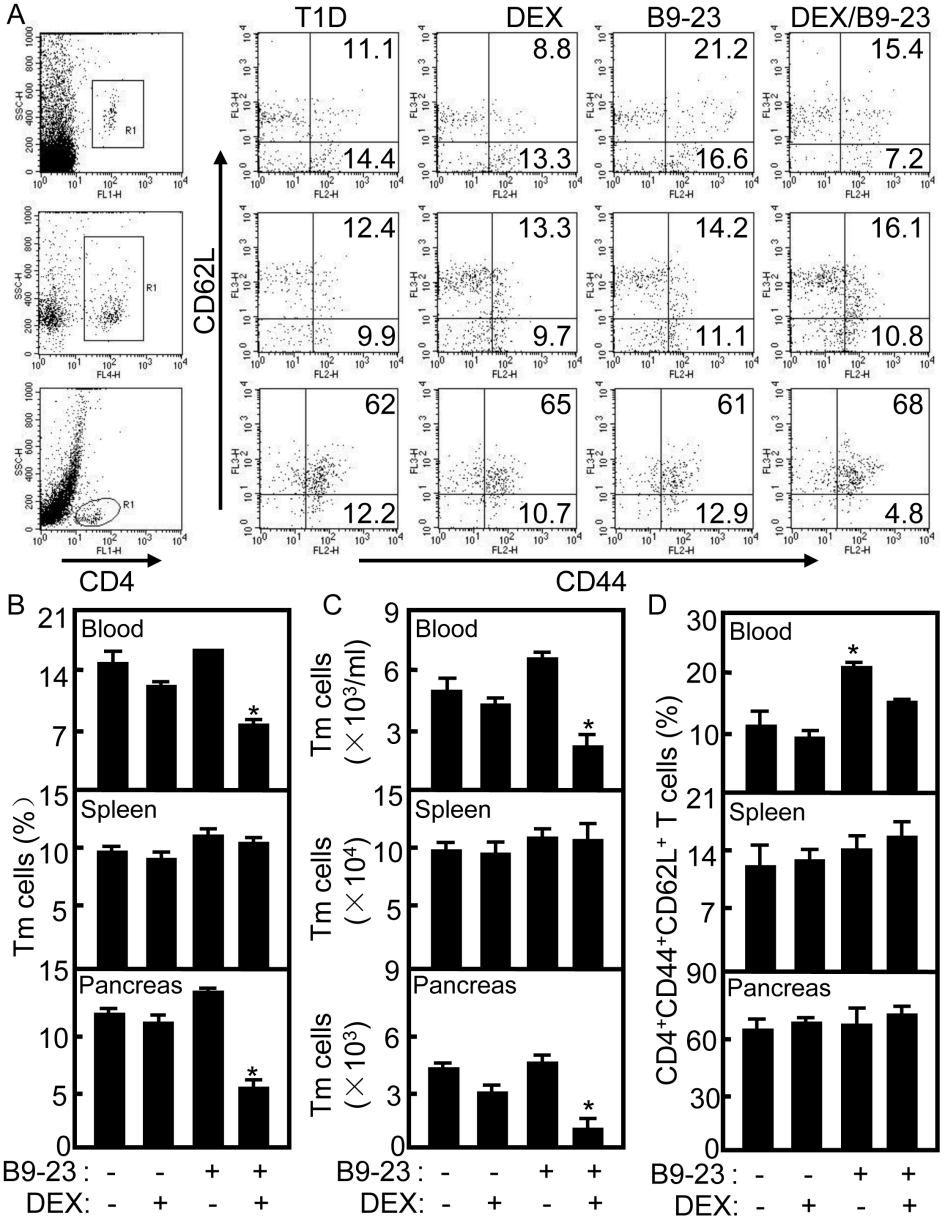
Suppression of effector memory CD4 T cells in treated diabetic mice. A. On day 45 after the second immunization, PBMC, splenocytes and pancreas cells were immunostained with anti-CD4-FITC, anti-CD62L-PECy5 and anti-CD44-PE mAbs and analyzed by flow cytometry. Gating on CD4^+^ T cell (R1), effector CD4 Tm cells (CD4^+^CD62L^-^CD44^+^) were counted relatively to total CD4 cells in PBMC (Upper), splenocytes (Middle) and pancreas cells (Lower). Shown in each panel is 1 of at least 3 experiments with similar results. For statistical analysis, mice treated with B9-23 were compared with that in other groups and ANOVA were used; *p<0.05. B. Summary of effector CD4 Tm cells in blood, in spleen or infiltrated in pancreases. Data shown are representative of 3 independent experiments. For statistical analysis, mice treated with B9-23/DEX were compared with other control groups and ANOVA were used; *p<0.05. C. The numbers of effector CD4 Tm cells in blood, in spleen or infiltrated in pancreases were counted by flow cytometry. Data shown are representative of 3 independent experiments. For statistical analysis, mice treated with B9-23/DEX were compared with other control groups and ANOVA were used; *p<0.05. D. The central memory CD4^+^CD62L^+^CD44^+^ T cells were counted relatively to total CD4 cells in PBMC (Upper), spleen (Middle) and pancreas (Lower). Shown in each panel is 1 of at least 3 experiments with similar results. For statistical analysis, mice treated with B9-23 were compared with that in other groups and ANOVA were used; *p<0.05.

When naive T cells are activated with antigen, the expression of several adhesion and homing molecules can be changed for effector Tm phenotype of CD44^high^, CD62L^low^ [7]. In T1D mice or patients, autoantigen-specific T cells have been shown to be antigen specific Tm cells whereas in healthy individuals [2]. To test whether the tolerogenic vaccination influence Tm cells in treated diabetic mice, the blood, splenic and pancreatic samples were prepared and stained for Tm cells on day 45 after the second treatment. Gating on CD4^+^ T cells (R1 in Figure 5A), effector memory CD4^+^CD44^+^CD62L^-^ T cells or central memory CD4^+^CD44^+^CD62L^+^ T cells were counted relatively to total CD4 cells. As shown in Figure 5B, the effector memory CD4^+^CD44^+^CD62L^-^ T cells in blood (Upper) were lowered significantly in B9-23/DEX treated mice compared with that in other groups (*p*<0.05) while there were no differences of CD4^+^CD44^+^CD62L^-^ T cells in spleen of all groups (Middle). Remarkably, the infiltrated CD4^+^CD44^+^CD62L^-^ T cells in pancreas (Lower) were lowered significantly in B9-23/DEX treated mice compared with that in other groups (*p*<0.05). Consistently, the number of central memory CD4^+^CD44^+^CD62L^-^ T cells was lowered significantly in blood and pancreas of B9-23/DEX treated mice compared with that in other groups (Figure 5C, *p*<0.05). The percentage of central memory CD4^+^CD44^+^ CD62L^+^ T cells was increased significantly in blood of B9-23 treated mice compared with that in other groups (Figure 5D, *p*<0.05). These results suggest that autoreactive effector memory CD4^+^CD44^+^ CD62L^-^ T cells were reduced in blood and pancreas of treated diabetic mice.

### Induction of Functional Effector Memory Treg Cells in Treated Diabetic Mice

**Figure 6:**
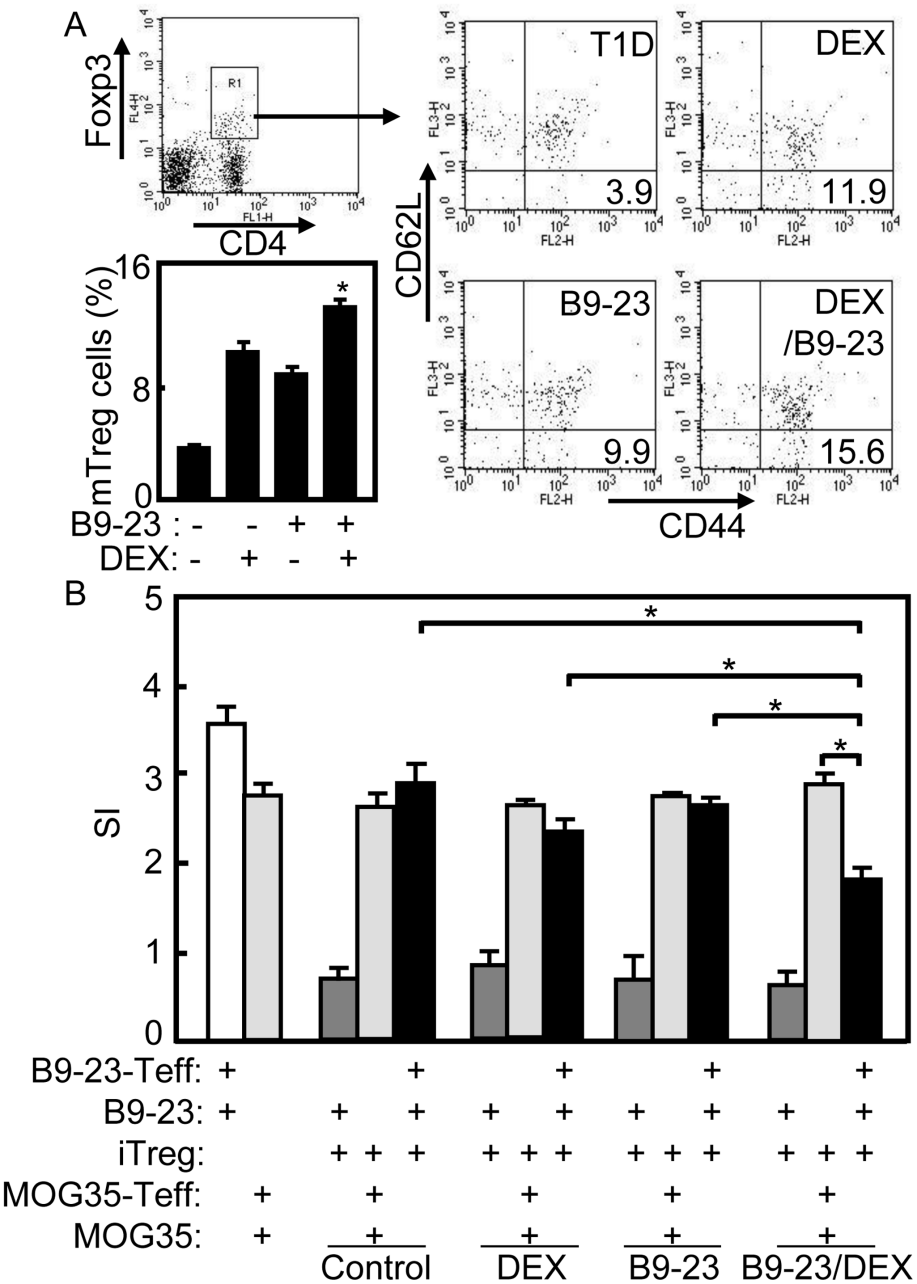
Generation of functional mTreg cells in treated diabetic mice. A. On day 45 after the second immunization, splenocytes were stained anti-CD4-FITC, anti-CD62L-PECy5, anti-CD44-PE mAbs and intracellularly stained withanti-Foxp3 -APC mAb, then analyzed by flow cytometry. Gating on Treg cells (CD4^+^Foxp3^+^), the effector mTreg cells (CD4^+^Foxp3^+^ CD44^+^CD62L^-^) were counted relatively to Treg cells. Shown in each panel is 1 of at least 3 experiments with similar results. Bar, mean and SD from 3 independent experiments, each using at least three mice per group (n = 3). For statistical analysis, mice treated with B9-23/DEX were compared with that in mice treated with B9-23 group and ANOVA were used; *p<0.05. B. On day 45 after the second immunization, Treg cells from diabetic mice treated with DEX/B9-23 were cocultured with Teff (CD4^+^CD25^−^) from mice immunized with IFA/B9-23 or IFA/MOG35-55, along with purified CD11c^+^ cells and B9-23 or IFA/MOG35-55. Proliferation was assessed by MTT method. Treg cells from mice of each group were purified respectively. Bar, mean and SD from 2-4 independent experiments, each using at least three mice per group (n = 3); For statistical analysis, mice treated with B9-23/DEX were compared with the indicated group and student-t test were used between the indicated pair; *p<0.05.

Naive Treg cells might be activated in the periphery by self-antigen and subsequently converted to mTreg cells in T1D mice or patients [28], [29]. The mTreg cells induced *in vitro* were capable of persisting as effector memory cells after transfer and were protective against the development of T1D [28], [30]. Several studies have reported the existence of a small population of Tregs and also mTreg cells in the peripheral blood of healthy adult individuals and preferentially activated Tm cells in diabetic patients [4], [29]. Since effector Tm cells appear phenotype of CD44^high^CD62L^low^, the CD4^+^Foxp3^+^ CD44^+^CD62L^-^ Treg cells were analyzed as effector memory Treg cells. On day 45 after the second treatment, the splenocytes of mice were prepared and immunostained for effector mTreg cells analysis by flow cytometry. Gating on Treg cells (CD4^+^Foxp3^+^, R1 in Figure 6A), the effector mTreg cells (CD4^+^Foxp3^+^ CD44^+^CD62L^-^) were counted relatively to total Treg cells. As shown in Figure 6A, the induced CD4^+^Foxp3^+^ CD44^+^CD62L^-^ effector mTreg cells were increased significantly in B9-23/DEX treated mice compared with that in other groups (*p*<0.05). The number of Treg cell in B9-23/DEX treated mice was higher than that in other groups, while the number of Treg cells in DEX treated mice was higher than that in T1D control and B9-23 treated groups (data not shown). This result suggests tolerogenic treatment stimulated the induction of CD4^+^Foxp3^+^ CD44^+^CD62L^-^ effector mTreg cells in treated diabetic mice. Additional experiments showed that the Treg cells from B9-23/DEX treated mice were functional and B9-23 specific, as they effectively inhibited the proliferation of B9-23 specific Teff and did not suppressed the proliferation of MOG35-55 specific Teff in coculture (*p*<0.05) (Figure 6B). These data established the capability of B9-23/DEX for induction of self-antigen specific CD4^+^Foxp3^+^ CD44^+^CD62L^-^ effector mTreg cells in treated diabetic mice.

## Discussion

T1D results from autoimmune destruction of insulin-producing β cells in the pancreatic islets. Once autoimmune responses are established, interventions are less effective on activated T cells, including Tm cells. Immunosuppressives that block activation and expansion of T cells have been used for T1D therapy [31]. Treg cells have been applied as strategies for prevention or therapy of autoimmune disease [2], [11], [28]. We previously demonstrated that DEX as adjuvant of B9-23 could induced and expanded Treg cells for T1D prevention [18]. The immune regulation of Treg cells has been studied, while their potential for developing immunological memory has received little attention. Here, we reveal that tolerogenic vaccination with B9-23/DEX reduced effector CD4 Tm cells and induced functional and specific effector mTreg cells for restoring immune tolerance for T1D treatment.

Vaccination with self-antigen or peptides induced immune tolerance by generating Treg cells for T1D prevention or therapy. Injections of GAD65_p217_ or GAD65_p290_ had no effect on T1D development in NOD mice [15]. This self antigen-based immunotherapy provides an approach to selectively tolerate self antigen-specific T cells, while keeping the remainder of the immune system intact [15]. When insulin protein was co-immunized with specific DNA plasmid, CD4^+^CD25^–^ islet-specific Treg cells were induced and effectively prevented T1D [17]. Otherwise, adjuvants as well as different routes of antigen administration can be used to manipulate the nature of the T cell response. Co-administration of IFA with insulin peptides subcutaneously protected T1D while intraperitoneal vaccination did not prevented T1D in NOD mice [16]. DEX can induce antigen-specific tolerance by influencing DC maturation, suppressing Th1 immune response, and promoting development of Treg cell [32]. Intraperitoneal injection of DEX into BALB/c mice for 1, 3, or 5 days enhanced the proportion of Treg cells in lymphoid organs, especially in the thymus [33]. Our previous study demonstrated B9-23/DEX vaccination could suppress established T cell responses by inducing Treg cells and expanded antigen-specific Treg for T1D prevention [18]. In this study, the proportion and number of Treg cells were consistently increased in B9-23/DEX treated diabetic mice which indicated this vaccination could induce antigen-specific Treg cells for T1D treatment. The B9-23 treatment could not induce Treg cells for T1D therapy which was similar with that in T1D prevention (Figure 2). Furthermore, the maturation of DCs was blocked in B9-23/DEX treated diabetic mice (Figure 3) which was consistent with that in tolerogenic vaccination for preventing T1D.

Many studies have demonstrated that CD4^+^ T cells play a predominant role in the development of insulitis, while CD8^+^ T cells migrate into the islets later and differentiate into killer cells with the aid of CD4^+^ T cells [34]. CD8 T cells from young NOD diabetic islets were able to transfer rapid onset of diabetes in NOD mice [35]. Others have demonstrated autoreactive T cells were preferentially activated in T1D patients [4]. Here, we also found autoreactive CD4 and CD8 T cell subsets in the pancreas of T1D mice. The CD4 T cells were decreased in the blood and pancreas of B9-23/DEX treated diabetic mice while there were no changes of CD8 T cells (Figure 4). Since the immunosuppressive function of DEX acts on T cells, the percentage of CD4 T cells in the blood, spleen and pancreas of DEX treated mice were decrease compared with that in T1D control group (Figure 4). In T1D mice and patients, the islet antigen specific T cells have already encountered and responded to the islet antigen, so it can convert to Tm cells and show memory phenotype [3]. Many studies have shown that CD44^High^CD62L^Low^ T cells appeared to be effector memory cells in T1D mice [6], [7], so the CD4^+^CD44^+^CD62L^-^ cells were used as effector Tm cells of mice on day 45 after treatment in this study. Here, the percentage and number of effector CD4 Tm cells were decreased significantly in blood and pancreas of B9-23/DEX treated diabetic mice compared with that in other control groups (Figure 5B) which indicate tolerogenic vaccination could function on effector memory cells. However, the phenotype and function of autoreactive Tm cells remains a challenge.

Treg cell as a biological therapy to restore self-tolerance may be a promising immune intervention for T1D [14]. However, this protective mechanism appears insufficient because of accumulation of pathogenic T cells over the long disease course [36]. Treg cells differentiated *in vitro* had acquired a typical memory phenotype that was maintained in NOD recipient mice, suggesting that Treg cells persisted in the hosts as effector memory cells [28], [30]. The mTreg cells could function in the long-term control of autoimmunity in T1D just as Tm cells have a role in the prevention of repeated infections and mTreg cells could use homeostatic mechanisms that are similar to conventional Tm cells [37]. Since dysregulation of Treg homeostasis appears characteristic of T1D, mTreg cells must utilize homeostatic mechanisms for long-term protection [8], and mTreg cells could be generated in T1D mice or patients [38], [39]. In this study, the percentage of effector mTreg cells were increased significantly in B9-23/DEX treated diabetic mice compared with that in other control groups (Figure 6A) suggesting the induction of effector mTreg cells. Importantly, these effector mTreg cells specifically suppressed the proliferation of effector T cells and showed potential to reestablish immune tolerance in T1D (Figure 6B).

In summary, our results demonstrate that tolerogenic vaccination effectively reduced effector CD4 Tm cells and induced effector mTreg cells for T1D treatment. Our findings provide an effective method for restoring tolerance by induction of effector mTreg, and may provide an attractive treatment for T1D.

## Materials and Methods

### Animals and Reagents

Female NOD mice aged at 6–8 weeks were purchased from Animal Institute of Chinese Medical Academy (Beijing, China). All animal protocols [#20120101] were approved by the Animal Welfare Committee of China Agricultural University and housed with pathogen-free food and water under 12 h light-cycle conditions. The B9-23 (SHLVEALYLVCGERG) peptide was from ChinaPeptides.Co, Ltd. DEX was from Sigma-Aldrich. The collagenase P was from Worthington. All antibodies for flow cytometry analysis were from eBioscience.

### NOD Mice Treatment and Immunization

The levels of glycemia of female NOD mice were determined weekly using glucose meter (Beijing Yicheng biological electronic technology Co., Ltd. JPS-6]. Mice tested positive (Glycemia level≧250 mg/dl) twice consecutively were used for treatment (n = 4). The diabetic mice were treated four times (on days 1, 4, 7, and 10) with DEX in the two hind footpads (16 µg/mouse). For the day-7 injection, B9-23 (2 µg/mouse) was coinjected with DEX. This regimen was given twice in a 2-wk interval. The levels of glycemia and death rate were checked weekly. Female non-diabetic NOD mice were immunized with IFA and insulin B9-23 or IFA and myelin oligodendrocyte glycoprotein peptide 35-33 (MOG35-55) twice in a 2-wk interval. On day 4 after the 2^nd^ immunization, the splenocytes were prepared as responsors for suppression assay.

### Histology Analysis

On day 7 after the second treatment, pancreases of treated NOD mice (n = 3) were collected and fixed in 4% formaldehyde for 24 h before being embedded in paraffin. Serial sections of 5 µm thickness were cut and stained with hematoxylin and eosin (H&E). Pancreatic sections from each group were scored blind for insulitis and insulitis was graded in at least 10 islets per pancreas: grade 0 - islet cells had no visible signs of inflammation; grade 1- the islets had lymphocytes surrounding the islet margin with little or no intraislet infiltration; grade 2 - islets were surrounded by lymphocytes and contained considerable intraislet inflammation; grade 3 - islets were completely engulfed with lymphocytes [40]. The mean insulitis score of each pancreas was calculated by dividing the sum of graded islets by the total number of islets analyzed.

### Immunostaining for Flow Cytometry

The blood and spleens of all groups (n = 3) were prepared and lysed to blood cells before staining for flow cytometry analysis. Pancreases of all groups were excised and cut into small pieces. The samples were digested with collagenase P (1 mg/ml) at 37°C in water bath and filtered with nylon net, then the samples were stained with mAbs for flow cytometry analysis.

For Treg cells analysis, the samples were intracellularly stained with anti-CD4-APC, anti-CD25-PECy5, and anti-Foxp3-PE mAbs. For IL-10 expression in Treg cells, the samples were stimulated in culture for 24 h with B9-23 and anti-CD28 mAb (eBioscience). The samples were treated with monensin (100 µg/ml) for 2 h and stained with anti-CD4-FITC, anti-CD25-PECy5 mAbs. The cells were fixed with 4% paraformaldehyde, permeabilized with 0.1% saponin, and then intracellularly stained with anti-Foxp3-APC mAbs and anti-IL-10-PE mAbs or isotype control (PE) of IL-10 mAb (eBioscience). Gating on CD4^+^ CD25^+^ T cells (R1), IL-10 expressing Treg cells (CD4^+^CD25^+^ Foxp3^+^ IL-10^+^) were quantified relatively to total Treg cells.

For DCs staining, the samples were stained with anti-CD11c-FITC, anti-MHCII-PE or anti-CD80-PE for DCs maturation. To detect IL-10 expression in DC, the splenocytes were stimulated with PMA (10 ng/ml), ionomycin (1 µg/ml) and monensin (2 µg/ml) for 4 h. After stimulation, the samples were washed and stained with anti-CD11c–FITC, fixed (4% paraformaldehyde), permeabilized (0.1% saponin), and intracellularly stained with anti-IL-10-PE mAb.

For T cells analysis, the samples were stained with anti-CD4-FITC and anti-CD8-APC mAbs. For effector memory CD4^+^ CD44^+^ CD62L^-^ T cell analysis, the samples were stained with anti-CD4-FITC, anti-CD44-PE and CD62L-PECy5 mAbs. For effector mTreg cells (CD4^+^Foxp3^+^CD44^+^ CD62L^-^) analysis, the samples were intracellularly stained with anti-CD4-FITC, anti-Foxp3-APC anti-CD44-PE and anti-CD62L-PECy5 mAbs. Gating on Treg cells (CD4^+^Foxp3^+^), the effector mTreg cells (CD4^+^Foxp3^+^ CD44^+^CD62L^-^) and the percentage of central memory Treg cells (CD4^+^Foxp3^+^ CD44^+^CD62L^+^) were counted relatively to Treg cells.

All the samples were analyzed with a FACScalibur and the Cell Quest Pro Software (BD Bioscience).

### Suppression Assay

CD4^+^CD25^-^ T effector (Teff) cells from NOD mice immunized with IFA/B9-23 (n = 3) were enriched via negative selection by magnetic cell sorting (Miltenyi Biotec, Auburn, CA), as per manufacturer’s protocols, and used as responders. Teff cells from mice immunized with IFA/MOG35-55 (n = 3) were also purified and used as responders for antigen specific control of Treg cells. CD4^+^CD25^+^ T cells from the spleen of treated diabetic mice (iTreg) were enriched via positive selection by magnetic cell sorting and used as suppressors while CD4^+^CD25^+^ T cells from the spleen of naïve NOD mice (nTreg) as control. CD11c^+^ cells were sorted by magnetic cell sorting (Miltenyi Biotec, Auburn, CA) from the spleen of naïve NOD mice and used as stimulators. The responders (1×10^5^ cells/well) were co-cultured with the suppressors (0.5×10^5^ cells/well), stimulators (1×10^4^ cells/well), and B9-23 (10 µg/ml) in U-bottom 96-well plates for 3 days at 37°C. MOG35-55 specific Teff cells were stimulated with MOG35-55 peptide (10 µg/ml) and co-cultured with the suppressors (0.5×10^5^ cells/well), stimulators (1×10^4^ cells/well) in U-bottom 96-well plates for 3 days at 37°C. The proliferation of the responder T cells was determined by the MTT method described before [41].

### Statistics

Results are depicted as mean±standard deviation (SD). Pairwise differences were analyzed by the two-sided Student’s t test. For multi-group analysis, ANOVA and the Bonferroni test were used. Differences are considered significant if *p*<0.05 and very significant if *p*<0.01.

## References

[1] Eisenbarth G (1986) Type I diabetes mellitus. A chronic autoimmune disease. N Engl J Med 314: 1360–1368. 3517648 10.1056/NEJM198605223142106

[2] Bluestone JA, Tang Q (2004) Therapeutic vaccination using CD4^+^CD25^+^ antigen-specific regulatory T cells. Proceedings of the National Academy of Sciences of the United States of America 101: 14622–14626. 15322272 10.1073/pnas.0405234101 PMC521996

[3] Viglietta V, Kent SC, Orban T, Hafler DA (2002) GAD65-reactive T cells are activated in patients with autoimmune type 1a diabetes. The Journal of Clinical Investigation 109: 895–903. 11927616 10.1172/JCI14114 PMC150925

[4] Danke NA, Yang J, Greenbaum C, Kwok WW (2005) Comparative study of GAD65-specific CD4^+^ T cells in healthy and type 1 diabetic subjects. Journal of Autoimmunity 25: 303–311. 16249070 10.1016/j.jaut.2005.08.007

[5] Zhao C, Davies JD (2010) A peripheral CD4+ T cell precursor for naive, memory, and regulatory T cells. The Journal of Experimental Medicine 207: 2883–2894. 21149551 10.1084/jem.20100598 PMC3005223

[6] Flynn J, McInerney M (2000) High density insulin receptor-positive diabetogenic T lymphocytes in nonobese diabetic mice are memory cells. Immunopharmacol Immunotoxicol 22: 387–400. 10952038 10.3109/08923970009016427

[7] Öling V, Reijonen H, Simell O, Knip M, Ilonen J (2012) Autoantigen-specific memory CD4^+^ T cells are prevalent early in progression to Type 1 diabetes. Cellular Immunology 273: 133–139. 22270037 10.1016/j.cellimm.2011.12.008

[8] Li C-R, Baaten BJG, Bradley LM (2012) Harnessing memory adaptive regulatory T cells to control autoimmunity in type 1 diabetes. Journal of Molecular Cell Biology 4: 38–47. 22116888 10.1093/jmcb/mjr040 PMC3269299

[9] Sakaguchi S (2004) Naturally arising CD4^+^ regulatory t cells for immunologic self-tolerance and negative control of immune responses. Annu Rev Immunol 22: 531–562. 15032588 10.1146/annurev.immunol.21.120601.141122

[10] Lobell A, Weissert R, Eltayeb S, de Graaf KL, Wefer J, et al (2003) Suppressive DNA Vaccination in Myelin Oligodendrocyte Glycoprotein Peptide-Induced Experimental Autoimmune Encephalomyelitis Involves a T1-Biased Immune Response. The Journal of Immunology 170: 1806–1813. 12574345 10.4049/jimmunol.170.4.1806

[11] Fontenot J, Gavin M, Rudensky A (2003) Foxp3 programs the development and function of CD4^+^CD25^+^ regulatory T cells. Nat Immunol 4: 330–336. 12612578 10.1038/ni904

[12] Shevach E (2002) CD4^+^ CD25^+^ suppressor T cells: more questions than answers. Nat Rev Immunol 2: 389–400. 12093005 10.1038/nri821

[13] Ferrera F, La Cava A, Rizzi M, Hahn B, Indiveri F, et al (2007) Gene vaccination for the induction of immune tolerance. Ann N Y Acad Sci 1110: 99–111. 17911425 10.1196/annals.1423.012

[14] Battaglia M, Roncarolo M-G (2011) Immune intervention with T regulatory cells: Past lessons and future perspectives for type 1 diabetes. Seminars in Immunology 23: 182–194. 21831659 10.1016/j.smim.2011.07.007

[15] Wang B, Tisch R (2008) Parameters influencing antigen-specific immunotherapy for type 1 diabetes. Immunologic Research 41: 175–187. 18543112 10.1007/s12026-008-8020-6

[16] Hutchings P, Cooke A (1998) Protection from Insulin Dependent Diabetes Mellitus Afforded by Insulin Antigens in Incomplete Freund’s Adjuvant Depends on Route of Administration. Journal of Autoimmunity 11: 127–130. 9650091 10.1006/jaut.1997.0184

[17] Zhang W, Jin H, Hu Y, Yu Y, Li X, et al (2010) Protective Response Against Type 1 Diabetes in Nonobese Diabetic Mice After Coimmunization with Insulin and DNA Encoding Proinsulin Human Gene Therapy. 21: 171–178. 10.1089/hum.2009.095 19788384

[18] Kang Y, Xu L, Wang B, Chen A, Zheng G (2008) Cutting Edge: Immunosuppressant as Adjuvant for Tolerogenic Immunization. The Journal of Immunology 180: 5172–5176. 18390698 10.4049/jimmunol.180.8.5172 PMC2377418

[19] Luo X, Tarbell KV, Yang H, Pothoven K, Bailey SL, et al (2007) Dendritic cells with TGF-β1 differentiate naïve CD4^+^CD25^−^ T cells into islet-protective Foxp3+ regulatory T cells. Proceedings of the National Academy of Sciences 104: 2821–2826. 10.1073/pnas.0611646104 PMC1815265 17307871

[20] Wong J, Mathis D, Benoist C (2007) TCR-based lineage tracing: no evidence for conversion of conventional into regulatory T cells in response to a natural self-antigen in pancreatic islets. The Journal of Experimental Medicine 204: 2039–2045. 17724131 10.1084/jem.20070822 PMC2118689

[21] Lo J, Peng R, Barker T, Xia C, Clare-Salzler M (2006) Peptide-pulsed immature dendritic cells reduce response to beta cell target antigens and protect NOD recipients from type I diabetes. Ann N Y Acad Sci 1079: 153–156. 17130547 10.1196/annals.1375.023

[22] Piemonti L, Monti P, Allavena P, Sironi M, Soldini L, et al (1999) Glucocorticoids Affect Human Dendritic Cell Differentiation and Maturation. The Journal of Immunology 162: 6473–6481. 10352262

[23] Dai Y, Carayanniotis G, Sercarz E (2005) Antigen processing by autoreactive B cells promotes determinant spreading. Cell Mol Immunol 2: 169–175. 16212883

[24] Gepts W, Lecompte P (1981) The pancreatic islets in diabetes. Am J Med 70: 105–115. 7006384 10.1016/0002-9343(81)90417-4

[25] Budd RC, Cerottini JC, Horvath C, Bron C, Pedrazzini T, et al (1987) Distinction of virgin and memory T lymphocytes. Stable acquisition of the Pgp-1 glycoprotein concomitant with antigenic stimulation. The Journal of Immunology 138: 3120–3129. 3106474

[26] Alleva DG, Crowe PD, Jin L, Kwok WW, Ling N, et al (2001) A disease-associated cellular immune response in type 1 diabetics to an immunodominant epitope of insulin. The Journal of Clinical Investigation 107: 173–180. 11160133 10.1172/JCI8525 PMC198872

[27] Kelemen K, Gottlieb PA, Putnam AL, Davidson HW, Wegmann DR, et al (2004) HLA-DQ8-Associated T Cell Responses to the Diabetes Autoantigen Phogrin (IA-2β) in Human Prediabetes. The Journal of Immunology 172: 3955–3962. 15004204 10.4049/jimmunol.172.6.3955

[28] Godebu E, Summers-Torres D, Lin MM, Baaten BJG, Bradley LM (2008) Polyclonal Adaptive Regulatory CD4 Cells That Can Reverse Type I Diabetes Become Oligoclonal Long-Term Protective Memory Cells. The Journal of Immunology 181: 1798–1805. 18641317 10.4049/jimmunol.181.3.1798 PMC3025323

[29] Monti P, Scirpoli M, Rigamonti A, Mayr A, Jaeger A, et al (2007) Evidence for In Vivo Primed and Expanded Autoreactive T Cells as a Specific Feature of Patients with Type 1 Diabetes. The Journal of Immunology 179: 5785–5792. 17947651 10.4049/jimmunol.179.9.5785

[30] Weber SE, Harbertson J, Godebu E, Mros GA, Padrick RC, et al (2006) Adaptive Islet-Specific Regulatory CD4 T Cells Control Autoimmune Diabetes and Mediate the Disappearance of Pathogenic Th1 Cells In Vivo. The Journal of Immunology 176: 4730–4739. 16585566 10.4049/jimmunol.176.8.4730

[31] Monfar M, Blenis J (1996) Inhibition of p70/p85 S6 kinase activities in T cells by dexamethasone. Molecular Endocrinology 10: 1107–1115. 8885245 10.1210/mend.10.9.8885245

[32] Franchimont D (2004) Overview of the Actions of Glucocorticoids on the Immune Response: A Good Model to Characterize New Pathways of Immunosuppression for New Treatment Strategies. Annals of the New York Academy of Sciences 1024: 124–137. 15265777 10.1196/annals.1321.009

[33] Chen X, Murakami T, Oppenheim JJ, Howard OMZ (2004) Differential response of murine CD4^+^CD25^+^ and CD4^+^CD25^–^ T cells to dexamethasone-induced cell death. European Journal of Immunology 34: 859–869. 14991616 10.1002/eji.200324506

[34] Yagi H, Matsumoto M, Kunimoto K, Kawaguchi J, Makino S, et al (1992) Analysis of the roles of CD4^+^ and CD8^+^ T cells in autoimmune diabetes of NOD mice using transfer to NOD athymic nude mice. Eur J Immunol 22: 2387–2393. 1516628 10.1002/eji.1830220931

[35] Wong FS, Visintin I, Wen L, Flavell RA, Janeway CA (1996) CD8 T cell clones from young nonobese diabetic (NOD) islets can transfer rapid onset of diabetes in NOD mice in the absence of CD4 cells. The Journal of Experimental Medicine 183: 67–76. 8551245 10.1084/jem.183.1.67 PMC2192404

[36] Tang Q, Bluestone JA (2006) Regulatory T-cell physiology and application to treat autoimmunity. Immunological Reviews 212: 217–237. 16903917 10.1111/j.0105-2896.2006.00421.x

[37] Li C-R, Deiro MF, Godebu E, Bradley LM (2011) IL-7 uniquely maintains FoxP3^+^ adaptive Treg cells that reverse diabetes in NOD mice via integrin-β7-dependent localization. Journal of Autoimmunity 37: 217–227. 21745722 10.1016/j.jaut.2011.06.002 PMC3431214

[38] Valmori D, Merlo A, Souleimanian NE, Hesdorffer CS, Ayyoub M (2005) A peripheral circulating compartment of natural naive CD4^+^ Tregs. The Journal of Clinical Investigation 115: 1953–1962. 16007258 10.1172/JCI23963 PMC1159133

[39] Fritzsching B, Oberle N, Pauly E, Geffers R, Buer J, et al (2006) Naive regulatory T cells: a novel subpopulation defined by resistance toward CD95L-mediated cell death. Blood 108: 3371–3378. 16868256 10.1182/blood-2006-02-005660

[40] Atkinson MA, Maclaren NK, Luchetta R (1990) Insulitis and Diabetes in NOD Mice Reduced by Prophylactic Insulin Therapy. Diabetes 39: 933–937. 2197139 10.2337/diab.39.8.933

[41] Kang Y, Zhao J, Liu Y, Chen A, Zheng G, et al (2009) FK506 as an adjuvant of tolerogenic DNA vaccination for the prevention of experimental autoimmune encephalomyelitis. The Journal of Gene Medicine 11: 1064–1070. 19688809 10.1002/jgm.1387

